# Glomus Tumor of the Stomach: A Rare Cause of Upper Gastrointestinal Bleeding

**DOI:** 10.1155/2011/371082

**Published:** 2011-10-29

**Authors:** Enzo Fabrício Ribeiro Nascimento, Fábio Piovezan Fonte, Roberta Laís Mendonça, Ronaldo Nonose, Carlos Alberto Fonte de Souza, Carlos Augusto Real Martinez

**Affiliations:** ^1^Department of General Surgery, São Francisco University Hospital, 09190-370 Bragança Paulista, SP, Brazil; ^2^São Francisco University Medical School, 09190-370 Bragança Paulista, SP, Brazil; ^3^Division of Pathology, Specialized Center of Pathological Diagnosis (CEDAP), 09190-370 Bragança Paulista, SP, Brazil; ^4^Post-Graduate Program in Health Sciences, São Francisco University, Rua Rui Barbosa, 255 Apto. 32, 09190-370 Santo André, SP, Brazil

## Abstract

*Introduction*. Glomus tumors (GTs) are benign neoplasm originating from the glomus body, commonly described in subungual region. The involvement abdominal is rare. Our aim is to describe a case of glomus tumor of the stomach that presented upper gastrointestinal bleeding. A 34-year-old woman was admitted with upper gastrointestinal bleeding and underwent an upper endoscopy that showed bleeding arising from an ulcerated lesion, treated by sclerosis therapy. A new endoscopy confirmed a submucosal lesion in upper portion of the stomach. During the laparotomy, a tumor at the upper anterior wall of gastric body was found and resected by a vertical gastrectomy. The pathological exam revealed hyperplastic smooth muscle fibers of the muscularis propria of the stomach wall, surrounded by hyaline stroma. The immunohistochemistry panel was positive for smooth muscle actin and type IV collagen, with low rate of mitosis studied by Ki-67 which allowed the final diagnosis of a gastric glomus tumor. *Discussion*. The majority of intraperitoneal glomus tumors occur in the stomach, and it is phenotypically similar to those localized in peripheral sites. Gastric GT generally is a benign tumor although it can be malignant and have the potential to metastasize. *Conclusion*. Even though gastric glomus tumor is rarely described, it should be considered as a possible cause of a major upper gastrointestinal bleeding.

## 1. Introduction

Glomus tumor (GT) is a rare mesenchymal neoplasm arising from glomus body a dermal arteriovenous shunt responsible for skin thermoregulation [1, 2]. It is a rare neoplasia representing approximately 2% of all soft tissue tumors and usually founded on extremities [2]. GT is commonly noted in the subungual finger extremities and rarely involving visceral organs, although tumors in the tympanum, mediastinum, trachea, kidney, uterus, vagina, and stomach have been described previously [3]. 

GT usually is a benign neoplasm, and the malignant variant is extremely rare with a few case reports in the literature [3, 4]. Smol'jyannikov wrote that the first GT of the stomach was described by Talijeva in 1928 and, since then, less than 200 cases have been published in the worlds' literature [3, 5, 6]. The preoperative differential diagnosis with other types of gastric mesenchymal tumors is difficult to be made [7]. Most of cases present with gastrointestinal bleeding and ulcer-like symptoms; however, exsanguinating gastrointestinal hemorrhage is rare [8]. The histopathological study using conventional techniques of staining often does not allow for accurate diagnosis, making the use of immunohistochemistry become an indispensable tool. 

The purpose of this paper is to present one patient who had a severe upper gastrointestinal bleeding due to a gastric GT treated successfully by a vertical gastrectomy, whose diagnosis was confirmed by immunohistochemical panel.

## 2. Case Report

**Figure 1:**
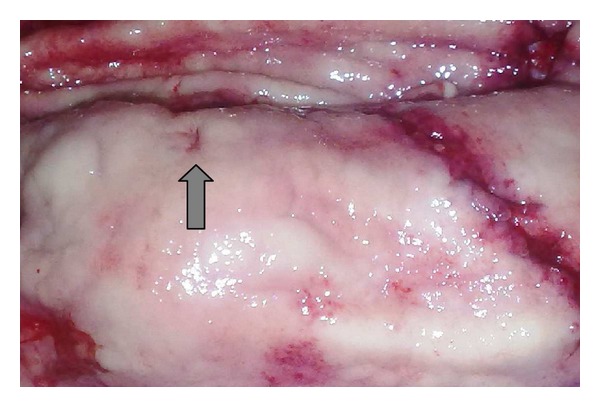
Submucosal tumor with a central ulceration (arrow).

**Figure 2:**
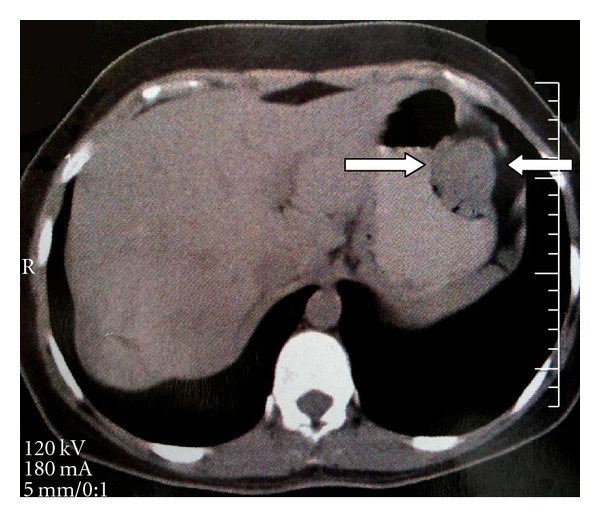
Glomus tumor of the stomach in a 34-year-old woman: on a contrast-enhanced computer tomography scan, the mass is greatly enhanced (arrows).

A 34-year-old woman was admitted with a massive upper gastrointestinal bleeding. She was hemodynamically unstable with signs of hypovolemic shock for which she required resuscitation with intravenous fluids and blood. Physical examination showed that the patient was confused, pale, and with cyanosis of extremities, tachycardia, and 70 × 40 mmHg of blood pressure. Family members reported that she had been complaining of epigastric pain, nauseas, weakness, and five episodes of melena in the previous three days. On physical examination, the abdomen was soft, nondistended, without palpable masses but slightly painful to deep palpation in the epigastric region. The digital rectal examination confirmed the presence of melena, and the hemoglobin level was 5.8 g/dL. After initial fluid recovery of the patient and the administration of 3U of blood concentrate, she underwent an emergency upper endoscopy. The exam showed an active bleeding arising from an elevated and ulcerated lesion located in the upper portion of the stomach at great curve. The bleeding was successfully controlled by local sclerosis therapy. Four days later, the patient was subjected to another upper gastrointestinal endoscopy that revealed mild, diffuse oesophagitis, and a small sliding hiatal hernia. At the cranial portion of the gastric corpus, a 5 cm, well-circumscribed reddish submucosal mass was observed (Figure 1). The gastric mucosa that covered the submucosal tumor showed a small ulcer, partially covered by fibrin, without signs of bleeding. Multiple regular biopsies were taken, and some histological features of smooth muscular tumor were identified. An abdominal CT scan confirmed the submucosal lesion which originated from the muscularis propria, measured 4.9 × 4.4 cm, and was compromising the longitudinal, muscular layers of the stomach that showed enhancement after the use of iodinated contrast (Figure 2). The exam showed no presence of hepatic metastasis or lymphadenopathy. 

**Figure 3:**
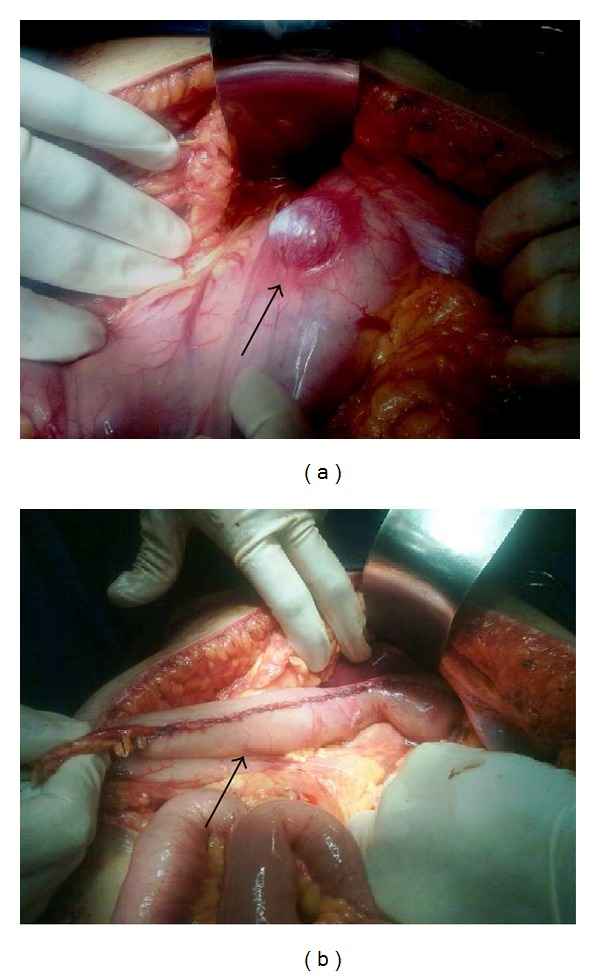
(a) The mass localized on anterior gastric wall with exuberant vascularization on serous surface. (b) Vertical gastrectomy along the great curvature.

With preoperative diagnosis of gastric leiomyoma, the patient was subsequently referred for elective surgical procedure. During the laparotomy, it was found a tumor measuring about 5 cm at its widest diameter, located in anterior gastric wall between the corpus and gastric fundus. The lesion infiltrated the gastric wall and compromised the serous layer where it was possible to observe large vascular proliferation (Figure 3(a)). The tumor was not adhered to the adjacent organs and was not identified hepatic or peritoneal metastasis. We had chosen to perform a vertical gastrectomy removing part of the anterior and posterior walls of the stomach along the greater curvature (Figure 3(b)). 

**Figure 4:**
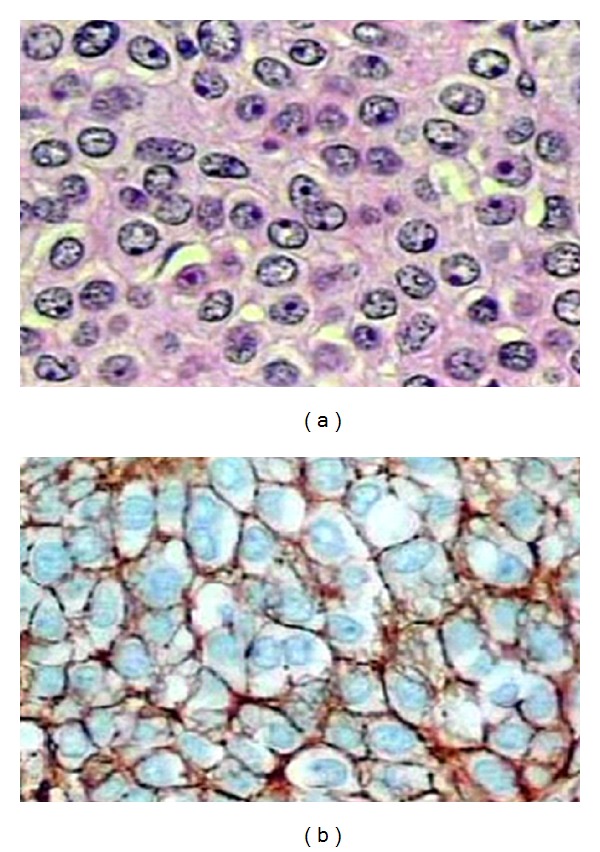
(a) Sheets of glomus cells with eosinophilic cytoplasm distributed around dilated blood vessels with stasis (H.E. ×1000). (b) Glomus tumor of the stomach. Positive staining for type IV Collagen (×1000).

The histopathological findings were characteristic of GT of the stomach. The cut surface of the specimen demonstrated a grayish-white nodular tumor, arising from the submucosa of the stomach and extending throughout all layers of the muscular propria, involving the serous surface. Histologically, the tumor was composed of sheets of glomus cells, without nuclear pleomorphism and no mitotic figures. The cells had eosinophilic and focally clear cytoplasm. The nuclei of these cells are round, with inconspicuous nucleoli. The cells contours were clear, and it was possible to identify dilated blood vessels among the cell blocks and areas of stromal hyalinization (Figure 4(a)). We could not identify vascular, lymphatic, or nervous invasion.

The immunohistochemical panel showed that the tumor cells were positive for smooth muscle actin and collagen type IV (Figure 4(b)) and negative for desmin, CD34, CD117, S-100 protein, creatin kinase, p53, chromogranin, and cytokeratins (Table 1). The proliferating marker Ki-67 was <5% confirming the low rate of mitosis in each histological field analyzed. The gastric margins are free of neoplastic compromise, but the mucosa showed erosive gastritis and positivity for *Helicobacter pylori*. Ten lymph nodes retrieved from the major omentum near the tumor were free of metastatic cells. The patient recovered uneventfully and was discharged 7 days after surgery. Currently, the patient is well 8 months after surgery, without signs of relapse or gastrointestinal bleeding.

## 3. Discussion

Gastric GT is a rare benign mesenchymal neoplasia arising from the neuromyoarterial glomus [9]. The glomus apparatus consists of three vascular components: an afferent artery separated from an efferent venule by convoluted channels [10]. They are commonly observed wherever arteriovenous anastomoses functioning without an intermediary capillary bed are present, are sensitive to temperature variation, and play a role in regulating arterial blood flow [4, 8]. Glomus tumors are commonly observed in the extremities and are rarely found in visceral organs_,_ but they have also been described in the bone and joints, skeletal muscle, soft tissue, thimpanus, mediastinum, trachea, kidney, uterus, and vagina [11]. The majority of intraperitoneal glomus tumors occur in the stomach, and a previous clinical pathological study showed that gastric GTs are phenotypically similar to those localized in peripheral sites [12]. Gastric GT generally is a benign tumor although it can be malignant and have the potential to metastasize [13].

The first case of GT located in stomach is credited to Talijeva in 1928 [5, 14], and since then few cases have been published [4]. The incidence of gastric GT is much less common than gastrointestinal stromal tumors (GISTs) with only 1 in 100 diagnosis of GISTs being gastric GT [7]. GT of the stomach has a marked predominance in females, and one recent review of 57 cases estimated that women are more affected on a ratio of 1.6 : 1 [7, 15, 16]. GTs usually occur in the fifth or sixth decade of life however, in two clinicopathologic studies among oriental population, the age of onset ranged from 30 to 68 and 28 to 79 years old, respectively [7, 14]. Generally, the diameter of gastric GT ranges from 0.8 to 21 cm, which is much larger than that in the distal extremities [11, 14]. 

GT tumors usually arise in the intramuscular layer and typically occur as a solitary submucosal nodule that most frequently affects the greater curvature, antrum, and pylorus. Rarely, they are multiple lesions compromising the lesser curve, anterior, and posterior wall of the corpus of the stomach [9, 17, 18]. The preoperative diagnosis of GT is difficult to be made. The gastric barium series show that GT appears as smooth submucosal mass with or without ulceration. The abdominal sonographic findings have been described as a hypoechoic mass in the third or fourth submucosal layers with internal heterogeneous echogenicity mixed with hyperechoic spots and lacking a capsule [19]. On CT, they manifest as well-circumscribed submucosal masses with homogeneous density on unenhanced study and may contain tiny flecks of calcifications. After contrast administration, these tumors show, as occurred in our patient, strong enhancement on arterial phase images and persistent enhancement on portal venous phase images. On MRI, gastric GT showed slightly hypointense on T1-weighted images and slightly hyperintense on T2-weighted images and is hypervascular and exhibits persistent enhancement after gadolinium administration [20]. However, it is important to note that all imaging techniques fail to differentiate GT from other stromal or mesenchymal lesions. The above-mentioned imaging features can also be seen with other mesenchymal tumors, such as neuroendocrine tumors, GIST, schwannoma, and mainly vascular tumors such as hemangioma and hemangiopericytoma which may show a similar pattern [9, 18].

The endoscopic biopsy is usually not helpful due to the intramural nature of the tumors. However, in the patient of this present paper, the removal of multiple fragments from a same site allowed the collection of neoplastic tissue from the interior of the mass leading to preoperative suspicion of a gastric leiomyoma. The endoscopic ultrasonography (EUS) usually show heterogeneous tumors between the submucosal and muscularis propria layer, and these aspects may be confused with malignant GIST or leiomyosarcoma, which is also represented by a heterogeneous tumor on EUS [4, 21]. It is possible that an accurate diagnosis can be made by endoscopic, ultrasound-guided, fine-needle aspiration (FNA) but, so far, only three articles have reported a preoperative definite diagnosis with FNA [22–24]. Unfortunately, it was not possible to refer our patient to EUS and FNA.

Histologically, GT was well circumscribed located in gastric submucosa or muscularis and comprised of glomus cells surrounding capillaries. The glomus cells were small, uniform, and round without nuclear pleomorphism, mitotic figures, or necrosis. The stroma showed hyalinization or myxoid change in some patients, and sporadic mast cells could be seen inside of the stroma. Immunohistochemistry is essential in the differential diagnosis of GT, and the immunohistochemical panel generally showed that GT tumor was strongly and diffusely positive for smooth muscle actin, vimentin and actin, calponin, type IV collagen, and laminin. In the patient of this present paper, the immunohistochemical panel showed positivity for smooth muscle actin, actin, and type IV collagen. Other markers, including desmin, cytokeratin (AE1/AE3b), S-100 protein, creatine kinase, C-KIT (CD-117), CD34, DOG1 protein (K9), chromogranin A, p53 protein (DO-7) and neuron specific enolase, were negative. Although gastric GT is usually benign, malignant behavior cannot be excluded. The following classification criterion was proposed for malignant GT: deep location and size more than 2 cm; presence of atypical mitotic figure; combination of moderate to high nuclear grade and mitotic activity (5 mitoses/50 high-power fields) [25]. Nevertheless, it should also be mentioned that the classification criteria has been established for superficial or deep soft tissue glomus tumors. However, the above-mentioned criteria should be used by convention for GT. 

Gastrointestinal bleeding with hematemesis/melena and epigastric discomfort, like the patient of this paper, the most two common initial symptoms/signs and can be life-threatening or lead to chronic anemia. The patient currently reported that she had never experienced an episode of gastrointestinal bleeding before, despite reporting dyspeptic symptoms for several years, and needed to continue the use of omeprazole. Operative intervention should be carefully planned in cases of gastric GT. The majority of the patients reported in the literature were operated. As gastric GTs are mesenchymal tumors with potential malignant behavior, wedge resection with negative margins should be the treatment of choice [26]. Enucleation is not recommended due to the high recurrence rates [27]. Recently, authors report the use of endoscopic submucosal enucleation [27]. In five patients, the tumors could be removed by endoscopic enucleation, but in one the operation had to be discontinued due to significant bleeding during the procedure [28]. In other series, gastric perforation occurred in one patient and was successfully managed with hemoclips [28]. No local recurrence was observed during followup. 

The choice of vertical gastrectomy was preferred by the team after the placement of surgical linear staplers around the lesion. As the location and extent of tumor resection with stapling wedge (wedge-resection) would result in significant gastric deformity with probable involvement of the organ and because vertical gastrectomy is a technique already realized and with good functional results, it was more appropriate.

There are few data in literature on postoperative followup of gastric glomus tumors; as the recurrence of these tumors usually occur at the site of removal of the tumor and are rare, we recommend performing endoscopy annually.

In conclusion, gastric GT is rare benign mesenchymal neoplasm and preoperative diagnosis is difficult. Since patients have no specific clinical and imaging findings, it is difficult to diagnose before operation. The differential diagnosis includes gastrointestinal stromal tumor, paraganglioma, and carcinoid tumor. The diagnostic gold standard for such lesions is the histological examination and the immunohistochemical markers. The correct approach of the patient optimizes the chances for an accurate preoperative diagnosis and leads to a targeted surgical or endoscopic intervention.

## Figures and Tables

**Table 1:** Antibodies, dilutions, sources, and results of immunohistochemical staining analysis of sections of a gastric glomus tumour in a 34-year-old woman.

Antibody	Dilution	Result
*α*-smooth muscle actin (HHF35)^a^	1 : 100	Positive
AE1/AE3b	1 : 200	Negative
Actin^b^	1 : 200	Positive
Collagen type IVb	1 : 400	Positive
S-100 protein^b^	1 : 400	Negative
Desmin^b^	1 : 3.200	Negative
Creatine kinase^b^	1 : 125	Negative
CD117^b^	1 : 1.1600	Negative
CD34^c^	1 : 50	Negative
DOG1 protein (K9)^b^	1 : 50	Negative
p53 protein (DO-7)^b^	1 : 200	Negative
Chromogranin A (DAK A-3)^b^	1 : 600	Negative
Ki-67 (MIB-1)^b^	1 : 4800	Positive (<5%)

^a^Thermo Fisher Scientific, Waltham, Mass, USA;

^b^Dako, Glostrup, Denmark, Calif, USA;

^c^Santa Cruz Biotechnology, Santa Cruz, Calif, USA; Novocastra, Newcastle, UK.
